# Cognitive and Emotional Factors Influencing the Incorporation of Advice Into Decision Making Across the Adult Lifespan

**DOI:** 10.1093/geronb/gbae080

**Published:** 2024-05-13

**Authors:** Tarren Leon, Gabrielle Weidemann, Ian I Kneebone, Phoebe E Bailey

**Affiliations:** Graduate School of Health, University of Technology Sydney, Sydney, New South Wales, Australia; School of Psychology, Western Sydney University, Sydney, New South Wales, Australia; MARCS Institute for Brain, Behaviour, and Development, Western Sydney University, Sydney, New South Wales, Australia; Graduate School of Health, University of Technology Sydney, Sydney, New South Wales, Australia; Graduate School of Health, University of Technology Sydney, Sydney, New South Wales, Australia; (Psychological Sciences Section)

**Keywords:** Advice-taking, Decision making, Depression, Emotion regulation, Judge–advisor

## Abstract

**Objectives:**

The present study sought to investigate the influence of advice on decision making in older age, as well as the potential influence of depressive symptoms and age-related differences in the cognitively demanding emotion regulation on advice-taking.

**Method:**

A nonclinical sample (*N* = 156; 50% female; 47 young: *M* age = 29.87, standard deviation [*SD*] = 5.58; 54 middle-aged: *M* age = 50.91, *SD* = 7.13; 55 older: *M* age = 72.51, *SD* = 5.33) completed a judge–advisor task to measure degree of advice-taking, as well as measures of fluid intelligence, depressive symptoms, confidence, perceived advice accuracy, and emotion regulation.

**Results:**

Age did not influence degree of advice-taking. Greater depressive symptoms were associated with more reliance on advice, but only among individuals who identified as emotion regulators. Interestingly, older age was associated with perceiving advice to be less accurate.

**Discussion:**

The study contributes to the sparse literature on advice-taking in older age. Cognitive and emotional factors influence the degree to which advice is incorporated into decision making in consistent ways across the adult lifespan. A key difference is that older adults take as much advice as younger adults despite perceiving the advice to be less accurate.

Conscious, analytical, and reason-based deliberative decision-making processes decline with age, while implicit and more automatic processes, based on intuition or affect, tend to remain intact (Peters et al., 2007). Relative to young adults, older adults are also less autonomous and more avoidant and dependent in their decision making (Mather, 2006). Löckenhoff (2018) suggested that this tendency toward dependent decision making in older age may reflect regulation of affect or limited cognitive resources. This is consistent with dual process models that acknowledge the roles of both cognition and emotion in decision making, while highlighting the need to investigate the potential interactions between these two processes (Peters et al., 2007). There is also preliminary evidence that, relative to young adults, older adults rely more on advice in their decision making (Bailey et al., 2021), which may be a form of dependent, or avoidant, decision making. This can be advantageous when advice is good quality but may not be beneficial when advice-taking occurs regardless of advice quality (Rader et al., 2017). The current study contributes to the limited evidence base for age-related differences in the degree of advice-taking (see Bailey et al., 2023 for a meta-analysis), while also investigating relative contributions of cognition and emotion to this decision-making process.

Dual process models of decision making acknowledge the influence of affect that is integral to the decision at hand, as well as decision-independent incidental affect (Peters et al., 2007). The current study focuses on incidental affect, while controlling for decision-related affect by using a simple decision-making task (i.e., estimating the number of coins in a jar) without any information about the advice-giver. Research has shown that incidental affect influences decision-making processes (see George & Dane, 2016), including the extent to which young adults rely on advice (de Hooge et al., 2014). For example, greater advice-taking was found among young adults with clinical depression, relative to controls (Hofheinz et al., 2017). There is also a strong positive correlation between avoidant decision-making and depressive symptoms (Leykin & DeRubeis, 2010).

Although previous research has not assessed the influence of depressive symptoms in older adults’ advice-taking, older adults with clinical depression make more disadvantageous selections in a repeated decision-making task in comparison to older adults without depression (Siqueira et al., 2021). In addition, among older adults, greater depressive symptoms were associated with decreased fluid intelligence (Aichele et al., 2019), and impairments in immediate recall (Murphy & O’Leary, 2010), processing speed, and executive functioning (Arnett & Vargas, 2010). Taken together, cognitive impairments associated with depressive symptoms may increase decision avoidance and dependence, including reliance on advice in older age.

Any influence of depressive symptoms on decision making in older age should be considered in the context of older adults having more effective emotion regulation abilities relative to young adults (Burr et al., 2021; You et al., 2019). Older adults have also been shown to devote more cognitive resources to repairing negative mood than to fully assessing all relevant elements of another task (Mienaltowski & Blanchard-Fields, 2005). Similarly, greater executive function impairment is demonstrated among older adults relative to young adults following a mood induction (Phillips et al., 2002). It was suggested that mood may create cognitive load for older adults or may decrease attention to other cognitive tasks. According to the selective-optimization-with-compensation model, older adults optimize their best skills, such as emotion regulation, to ensure efficient use of limited cognitive resources (Baltes & Baltes, 1990). It is therefore possible that older adults with depressive symptoms will direct cognitive resources away from decision making in favor of emotion regulation. Alternatively, age-related changes in cognitive capacity may influence the extent to which older adults engage in a particular emotion regulation strategy.

Although all types of emotion regulation rely on some degree of cognitive capacity, active emotion regulation involves more effortful attempts to directly influence emotion (Coats & Blanchard-Fields, 2008), whereas passive emotion regulation reduces reliance on cognition (Allen & Windsor, 2019; Blanchard-Fields et al., 2004). Compared with adolescents and young adults, older adults use more passive-based (i.e., acceptance) strategies in their general emotion regulation toward stressful life events (Garnefski & Kraaij, 2006). Additionally, older adults’ use of a passive strategy was positively related with greater depressive symptom scores. The present study therefore explored whether recent attempts to accept (a passive strategy), relative to change (an active strategy) emotion, differentially influenced the association between older adults’ depressive symptoms and advice-taking.

The present study sought to investigate mechanisms underpinning decision making in a nonclinical adult lifespan sample, and particularly the influence of depressive symptoms on older adults’ advice-taking from an unknown source. While advice can be framed as coming from different sources (e.g., experts or novices), this can have its own effect on advice-taking (Meshi et al., 2012). Thus, the present study controlled for characteristics of the advisor. The first hypothesis was that with increasing age, and greater depressive symptoms, there would be greater advice-taking. That is, depressive symptoms were expected to moderate the strength of the relationship between age and advice-taking in a cross-sectional sample. Second, it was predicted that greater depressive symptoms among older adults would be associated with lower fluid intelligence, and that this association may be mediated by whether the individual tends toward being an emotion regulator (i.e., has recently attempted to change or accept their emotion) relative to a nonregulator. In either case, we expected that lower fluid intelligence would be associated with greater advice-taking. Exploratory analyses examined whether a tendency toward being an active (i.e., change-focused) versus passive (i.e., acceptance-focused) emotion regulator differentially influenced the association between depressive symptoms and advice-taking. We also explored associations between age, confidence, emotion regulation effort, type of emotion regulation (accept or change), perceptions of advice accuracy, and advice-taking. These exploratory analyses were not preregistered.

## Method

### Participants

An a priori power analysis using G*Power (Faul et al., 2007) showed that to detect a small- to medium-sized effect (*f* = 0.15) of age on advice-taking (Bailey et al., 2021) for a multiple linear regression with six predictors, α = 0.05 and 95% power, 146 participants (i.e., rounded up to 50 young, 50 middle-aged, and 50 older) were required. Participants were excluded if they reported a neurological condition (such as Alzheimer’s disease, stroke, or brain injury, or neuroatypical disorders). Participants were not excluded if they reported current or previous major depressive disorder. One hundred and sixty-six Australian resident participants were recruited online via *Qualtrics Panels*. Additional participants were included by Qualtrics to account for potential exclusions. Data for 10 participants (young group: one male, four female; middle-aged group: three female; older group: one male, one female) were removed from analyses due to reporting a current neurological condition. The final sample consisted of 47 young (*M* age = 29.87 years, standard deviation [*SD*] = 5.58; range = 19–38; 25 female; average years of education: 15.09), 54 middle-aged (*M* = 50.91 years, *SD* = 7.13; range = 40–64; 25 female; average years of education: 14.52), and 55 older adults (*M* = 72.51 years, *SD* = 5.33; range = 65–88; 28 female; average years of education: 12.89). Race and ethnicity of the sample was not recorded. However, U.S.-based studies have reported Qualtrics samples to be predominantly White and heteronormative (Douglas et al., 2023; Peer et al., 2022). All participants gave written informed consent, and the research was approved by the University of Technology Sydney Human Ethics Committee, approval ETH22-6857. The study was preregistered via *AsPredicted* (#103223), https://aspredicted.org/2DT_WSF. Data are available at https://osf.io/ymun4/?view_only=502d01b6c15c4389ab548b0038cd87a.

### Materials and Procedure

#### Judge–advisor task

The judge–advisor task provides a measure of advice-taking (Sniezek & Buckley, 1995). Participants provide initial quantitative estimates, receive numeric advice, and then provide revised estimates. Following Gino et al. (2012), participants estimated the number of coins in a jar. This was to equate base-level knowledge between different age groups, as was confirmed by our pilot study (*N* = 132; age range = 19–89 years).

**Figure 1.**
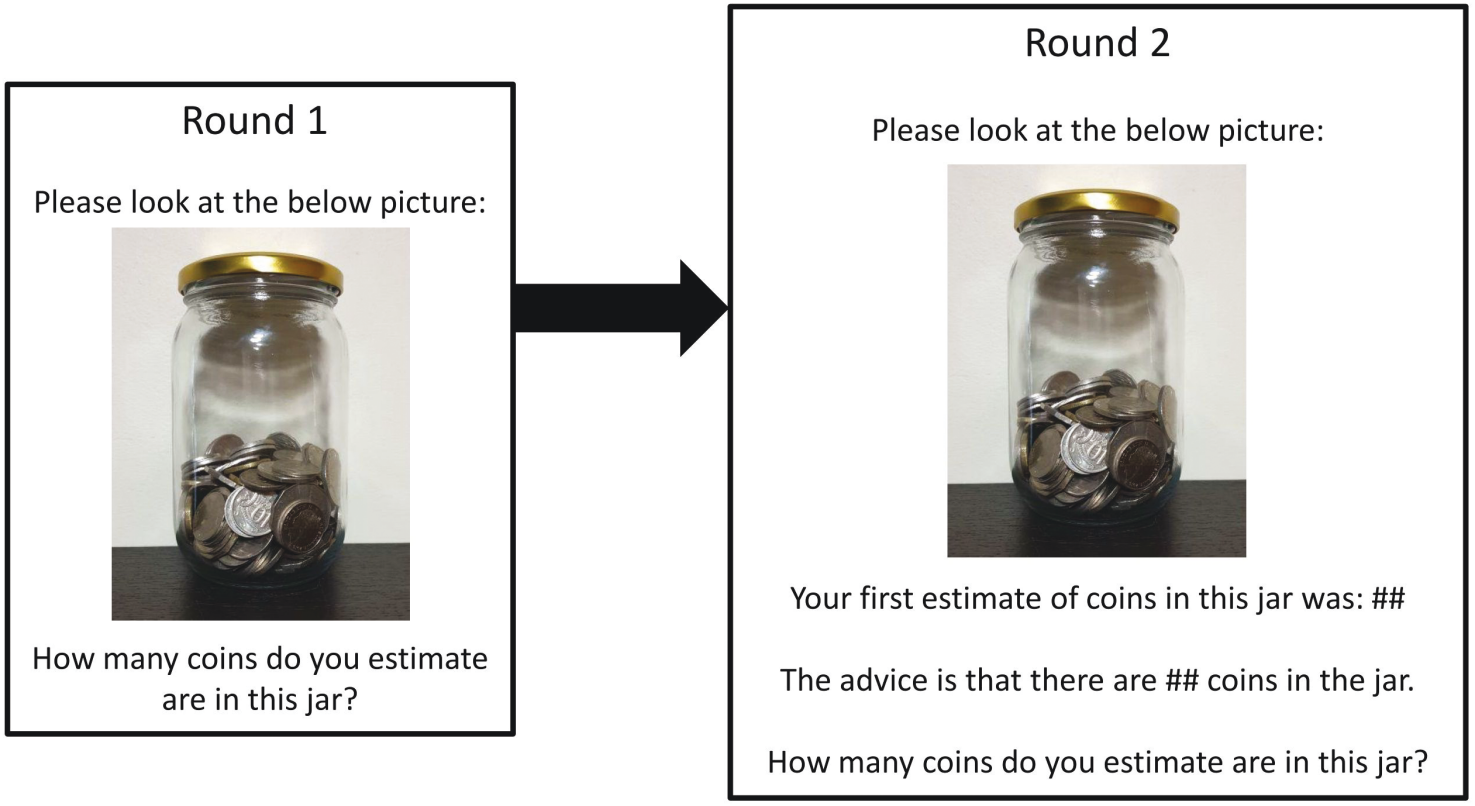
Judge–advisor task instructions for Rounds 1 and 2.

Prior to the main task, each participant completed three practice trials without and with advice (see Supplementary Material Section 1 for more information). In the Round 1 of the main judge–advisor task, participants viewed 12 images of jars of coins, one at a time, and estimated the number of coins in each jar (range = 36–273 coins). The order of image was randomized for each participant. In Round 2, participants saw the same 12 images, one at a time in random order, along with their own initial estimate and the advice, which was not framed as coming from a particular source. To control for the influence of the advisor, no advisor characteristics were revealed. Refer to Figure 1 for an illustration of task progression and instructions.

Prior to Round 2, participants were told, “You will view the same set of 12 jars that you saw earlier. This time you will be shown your first estimate for each jar, as well as some advice. You can then make another estimate of the number of coins in the jar. Please try your best.” Participants were unaware that the advice was always within ±5% of the correct number. After providing their second estimate, participants were not provided any feedback regarding the accuracy of their estimate. Reliance on advice is measured using the *weight of advice* calculation: [(final estimate − initial estimate)/(advice − initial estimate)]. A weight of advice score of 0 indicates no reliance on advice, while a score of 1 indicates complete reliance on advice. Refer to Supplementary Material Section 1 for a description of data preparation and cleaning.

#### Depressive symptoms

Current levels of depressive symptoms were measured using the depression subscale of the Depression Anxiety and Stress Scale-21 (DASS-21; S. H. Lovibond & Lovibond, 1995). The subscale includes seven items, rated on a 4-point scale from 0 (“did not apply to me at all”) to 3 (“applied to me very much, or most of the time”), to indicate how much each statement applied to participants over the past week. Items included “I couldn’t seem to experience any positive feeling at all” and “I felt down-hearted and blue.” The DASS-21 is appropriate for use among nonclinical samples (P. F. Lovibond & Lovibond, 1995), and has been validated with samples of older adults (Gholamzadeh et al., 2019; Gloster et al., 2008; Thapa et al., 2020). Higher scores indicate elevated depressive symptoms on a continuum of severity of symptoms in the population (S. H. Lovibond & Lovibond, 1995). Cronbach’s alpha for the present sample was 0.95.

#### Emotion regulation

To assess recent emotion regulation strategies, participants were first asked if they had done anything in the last 2 weeks to change or influence their feelings. They were advised that this could include “entering or avoiding a situation or person”; “shifting attention to certain aspects of a situation”; “changing their thinking, and/or changing their emotional expression” (adapted from Livingstone & Isaacowitz, 2021). If participants answered “no,” they were asked if they had experienced any strong positive or negative emotions in the past 2 weeks, and were given the response options “no” and “yes, I accepted them/let them play out.” If they answered yes to either changing or accepting their emotions, they were asked to indicate how mentally effortful this was, on a scale from 0 (not effort at all) to 10 (the most effort). In addition to the index of regulation effort, participants were classified as emotion regulators or nonregulators based on answering “yes” to changing or accepting emotions versus answering “no” to trying to influence their feelings, respectively.

#### Fluid intelligence

Letter sets (Ekstrome et al., 1976) and number series (Thurstone, 1938) tasks assessed fluid intelligence. The versions used in the current study have been validated in previous research (Foster et al., 2014; Hicks et al., 2016). In the letter sets there were 10 questions and participants were allowed a maximum of 5 min to complete the task. Participants were provided with one practice question before completing the 10 letter sets. The letter sets required participants to select which letter set did not fit the other options. There were 15 questions in the number series and participants had 3 min and 30 s to answer. Two practice questions were provided beforehand. The number series required participants to select the correct number (out of five options) that would come next in a given sequence. Higher scores averaged across the two tasks indicated better performance.

#### Self-confidence

At the end of each judge–advisor round (Round 1 = confidence ratings when no advice had been received [i.e., pre-confidence]; Round 2 = confidence ratings after receiving advice [i.e., post-confidence]), participants rated how confident they were in their own estimates on a scale from 1 (not at all confident) to 5 (very confident). Participants also rated how accurate they thought the advice was on a scale of 1 (not at all) to 5 (very).

## Results

See Supplementary Material Section 2 for software and packages used for analyses and Table 1 for descriptive statistics for each measure as a function of age group.

**Table 1.** Descriptive Statistics for Scales and Tasks as a Function of Age Group

Variable	Young adults			Middle-aged adults			Older adults		
	*M*	*SD*	*n*	*M*	*SD*	*n*	*M*	*SD*	*n*
Pre-confidence	2.94	1.13		2.78	0.98		2.58	0.98	
Post-confidence	3.21	1.12		3.11	1.06		2.69	1.07	
PAA	3.55	0.78		3.20	1.05		2.82	0.98	
DSS	6.96	6.09		6.13	5.31		2.97	4.12	
Fluid IQ	4.01	2.07		4.14	1.76		4.00	1.56	
Average WOA	0.63	0.25		0.67	0.31		0.62	0.26	
ER effort	6.66	2.07		6.85	1.92		5.32	2.17	
ER type									
None			9			21			21
Accept			13			12			16
Change			25			21			18

*Notes*: DSS = depressive symptom score; ER = emotion regulation; PAA = perceived advice accuracy; IQ = average fluid intelligence score; *SD* = standard deviation; average WOA = the average weight of advice across the 12 trials.

### Practice Phase

There was no age group by practice trial interaction, suggesting base-level knowledge was equated across age. See Supplementary Material for further details.

### Primary Analyses

Where mixed-effects models were conducted in the following analyses, continuous fixed-effects predictors were grand mean-centered, as this is appropriate for between-subjects analyses (Enders & Tofighi, 2007). Baseline of continuous predictor variables are the average values for that variable (i.e., age β estimates would indicate the change in the outcome variable per 1-unit change from the average age). Model selections were determined by log-likelihood ratio test results, which are useful for simple comparisons of nested models (Meteyard & Davies, 2020). Where Q–Q plots suggested nonnormality, robust mixed-effects models were used. Such models can be suitable for controlling nonnormality and reducing any residual outlier contamination (Koller, 2016).

#### Age, depressive symptoms, and advice-taking

To assess our first hypothesis regarding the effects of age and depressive symptoms on advice-taking, mixed-effects models were created. Weight of advice was set as the outcome, and fixed-effects predictors consisted of continuous measures of age and depressive symptoms score. Participant ID was set as a random effect. Two models were created and compared against each other, one with an interaction between the two fixed effects, and one without.

There were no significant effects of age, β = 0.00, standard error (*SE*) = 0.00, 95% confidence interval (CI) [−0.00, 0.00], *t*(151) = 0.51, *p* = .612, or depressive symptoms, β = 0.01, *SE* = 0.00, 95% CI [−0.00, 0.00], *t*(156) = 1.19, *p* = .238, on advice-taking. The model with age and depressive symptoms interaction did not improve model fit (as per Supplementary Table 3). The final model results are reported in Supplementary Table 4.

#### Correlations

To assess our second and third hypotheses, we first examined correlations between age, depressive symptoms, fluid intelligence, emotion regulation (change/accept vs none), and advice-taking. We also included exploratory variables (pre- and post-advice confidence, and perceived advice accuracy) in the correlation matrix. Table 2 shows that age was negatively correlated with depressive symptoms, emotion regulation, post-advice confidence, and perceived advice accuracy (*r*s ≥ −0.18, *p*s ≤ .021). Depressive symptoms were positively correlated with emotion regulation and perceived advice accuracy (*r*s ≥ 0.27, *p*s ≤ .002), and negatively associated with fluid intelligence (*r* = −0.22, *p* = .006). Emotion regulation was positively correlated with post-advice confidence, and perceived advice accuracy (*r*s ≥ 0.21, *p*s ≤ .008). Perceived advice accuracy had a negative relationship with fluid intelligence (*r* = −0.18, *p* = .021), and a positive relationship with the average weight of advice (*r* = 0.24, *p* = .003).

**Table 2.** Intercorrelations Between Age, Depressive Symptoms, Pre- and Post-Advice Confidence, Perceived Advice Accuracy, Fluid Intelligence, and Average Weight of Advice Among All Participants (*N* = 156)

Variable	1	2	3	4	5	6	7	8
1. Age	—							
2. DSS	−0.36***	—						
3. ER	−0.18*	0.27***	—					
4. Pre-confidence	−0.13	−0.02	0.07	—				
5. Post-confidence	−0.21**	0.11	0.21**	0.68***	—			
6. PAA	−0.28***	0.25**	0.25**	0.35***	0.49***	—		
7. Fluid IQ	0.05	−0.22**	−0.11	−0.15	−0.09	−0.18*	—	
8. WOA	0.02	0.07	0.07	−0.14	−0.09	0.24**	−0.03	—

*Notes*: DSS = depressive symptom score; ER = emotion regulator (0 = no, 1 = yes—change/accept); IQ = average fluid intelligence score; PAA = perceived advice accuracy; WOA = average weight of advice.

*p < .05. **p < .01. ***p < .001.

Because age, depressive symptoms, emotion regulation, and fluid intelligence were not correlated with weight of advice (all *r*s ≤ 0.15, all *p*s ≥ .05), we did not analyze whether being an emotion regulator mediated an association between depressive symptoms in older age and weight of advice, nor whether confidence mediated an association between depressive symptoms and weight of advice.

### Exploratory Analyses

#### Emotion regulation method and advice-taking

Separate correlational analyses between age, depressive symptoms score, emotion regulation effort, confidence, perceived advice accuracy, average fluid intelligence score (IQ), and weight of advice were conducted for each of the emotion regulation methods (change method [see Supplementary Table 7], acceptance method [see Supplementary Table 8], and emotion nonregulator [see Supplementary Table 9]). Results are reported with Benjamini and Hochberg (1995) adjustments. See Supplemental Material for the full results relating to self-confidence.

##### Change method of emotion regulation

As can be seen in Supplementary Table 7, there was a negative relationship between age and perceived advice accuracy (*r* = −0.38, *p* = .017). A positive association was found between depressive symptoms and effort exerted to change emotions (*r* = 0.38, *p* = .017). No other correlations among the variables were found (all *r*s ≤ 0.28, all *p*s ≥ .05).

##### Acceptance method of emotion regulation

As shown in Supplementary Table 8, there was a negative relationship between age and depressive symptoms (*r* = −0.47, *p* = .028). No other relationships among the variables were found (all *r*s ≤ 0.31, all *p*s ≥ .05).

##### Nonregulator

As can be seen in Supplementary Table 9, among those who did not report any emotion regulation, there was a positive relationship between depressive symptoms and average weight of advice (*r* = 0.44, *p* = .016). No other relationships among the variables were found (all *r*s ≤ 0.35, all *p*s ≥ .05).

Given the correlation analyses indicated some differences in relationships between variables depending on the type of emotion regulation typically used, further mixed model analyses were conducted. Predictors were the emotion regulation method (change, acceptance, or none), age, depressive symptoms score, fluid IQ, pre-confidence, and perceived advice accuracy. The outcome variable was weight of advice. Interactions between the emotion regulation method and other predictor variables were examined. Model comparisons can be seen in Supplementary Table 10. Models with interactions between age, pre-confidence, and fluid IQ were also included to investigate any relationships among these variables.

**Figure 2.**
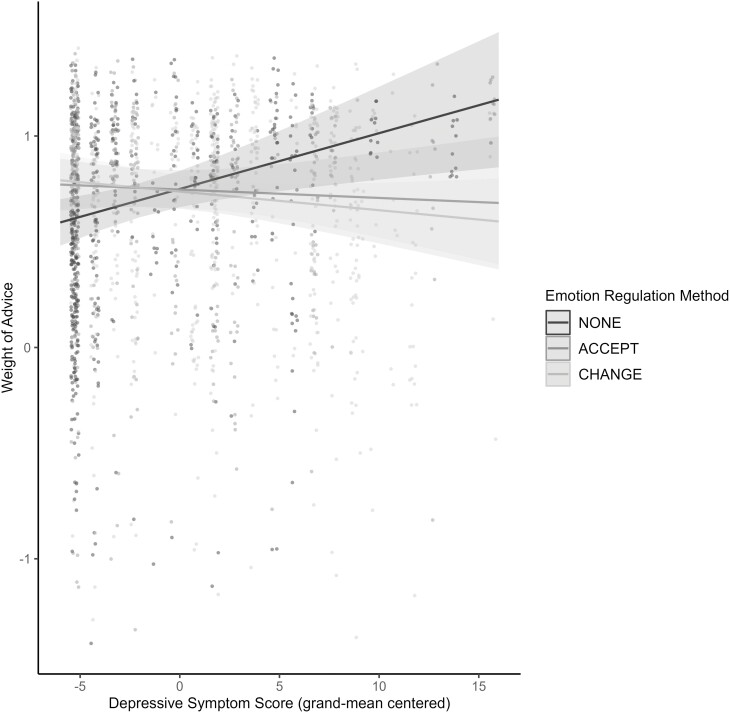
Graph of model interaction between depressive symptom score, and emotion regulation method, on weight of advice.

Due to violations of normality and linearity, a robust model was used, which can be viewed in Supplementary Table 11. The final model revealed two negative interaction effects between depressive symptoms and the change emotion regulation method (β = −0.04, *SE* = 0.01, 95% CI [−0.06, −0.01], *t*(148) = 3.19, *p* = .001), as well as depressive symptoms and the accept emotion regulation method (β = −0.03, *SE* = 0.01, 95% CI [−0.05, −0.01], *t*(146) = 2.47, *p* = .014), on weight of advice. One unit increase from the average depressive symptom score, along with having used the emotion regulation change or accept methods (as opposed to no emotion regulation method), had negative effects on advice-taking, as seen in Figure 2. The model also indicated a positive effect of depressive symptoms on weight of advice (β = 0.03, *SE* = 0.01, 95% CI [0.01, 0.04], *t*(146) = 2.96, *p* = .003), and a positive effect of perceived advice accuracy ratings on weight of advice (β = 0.11, *SE* = 0.03, 95% CI [0.06, 0.17], *t*(146) = 4.30, *p* < .001).

## Discussion

The overall aim of the current study was to better understand the influence of cognition and emotion on advice-taking in older adults’ decision making. The data did not support the first hypothesis that depressive symptoms would moderate an association between age and advice-taking. There was also no support for the second hypothesis that being an emotion regulator versus a nonregulator would mediate an association between greater depressive symptoms in older age and lower fluid intelligence. Our findings indicated that depressive symptoms were negatively associated with fluid intelligence regardless of age. There was, however, no association between emotion regulation effort and fluid intelligence for those engaging in the either the change or accept emotion regulation strategies, suggesting that emotion regulation may not have been responsible for the cognitive impairment. While the results did not support the third hypothesis that lower self-confidence would mediate an association between greater depressive symptoms and advice-taking, exploratory analyses revealed that pre-advice confidence had a negative influence on degree of advice-taking (as per Supplementary Table 11).

A novel finding was that, averaged across age, greater depressive symptoms were associated with greater advice-taking among individuals who reported being nonregulators of emotion, relative to those reporting recent use of either a change or acceptance method of emotion regulation. Unexpectedly, degree of advice-taking remained consistent across the adult lifespan sample, even though exploratory analyses revealed that older adulthood was associated with perceptions of advice accuracy as being lower.

### Depressive Symptoms, Emotion Regulation, and Advice-Taking

Previous research has shown that clinical depression in young adults is associated with greater advice-taking (Hofheinz et al., 2017). The current study provides new insights by demonstrating an association between greater depressive symptoms and more weight given to advice in an adult lifespan sample that includes older adults. More specifically, the association was evidenced among nonregulators of emotion, relative to those who reported recent engagement with emotion regulation. This finding is counter to the expectation that greater advice-taking in the context of depressive symptoms would occur due to redeployment of cognitive resources toward the regulation of depressive symptoms. One possibility is that depressive symptoms are directly associated with cognitive decline in some individuals (Aichele et al., 2019; Beaujean et al., 2013), and this not only increases advice-taking, but also prevents engagement with an emotion regulation strategy. Alternatively, engaging in emotion regulation may reduce cognitive decline associated with depressive symptoms, which lowers reliance on advice. Further research is required to test these different causal possibilities.

According to the selective-optimization-with-compensation model, and previous studies, older adults prioritize emotion regulation over other cognitive tasks (Baltes & Baltes, 1990; Mienaltowski & Blanchard-Fields, 2005; Phillips et al., 2002). It was speculated that increased depressive symptoms, which are associated with lower fluid intelligence, may result in older adults dedicating cognitive resources to mood regulation rather than other tasks such as the decision-making task. However, the data did not support any association between cognition and advice-taking among older adults. Additionally, there was no decrease in fluid intelligence scores with age.

### Self-Confidence and Perceived Advice Accuracy

Although pre-advice confidence was not correlated with advice-taking, the exploratory mixed-effects model revealed a negative influence of pre-advice confidence on advice-taking (Supplementary Table 11). This association did not interact with depressive symptoms, which contrasts with a previous finding that lower confidence mediated the relationship between increased anxiety and greater advice-taking (Gino et al., 2012). Pescetelli et al. (2021) reported a positive relationship between advice-taking and confidence ratings when the stimulus provided useful evidence that potentially led to advice being treated as more valid. However, the current data were more consistent with Hütter and Fielder’s (2019) finding that lower confidence was associated with greater advice-taking, possibly suggesting that participants were uncertain of their estimate.

Perceiving advice as more accurate was a predictor of greater advice-taking in the current study. We also found that older age was associated with lower perceived accuracy of advice, although age did not correlate with advice-taking or pre-advice confidence ratings. That is, older adults gave as much weight to advice as younger adults despite perceiving the advice to be of lower quality. This is broadly consistent with a previous study showing an age-related increase in the weight of advice in response to an advisor labeled as a novice, but no difference in response to an advisor labeled as an expert (Bailey et al., 2021). Perceived advice accuracy is the strongest predictor of advice-taking in the judge–advisor paradigm (Bailey et al., 2023), and accuracy is partly predicted by trust, even when advisor attributes are unknown (Wang & Du, 2018). It will therefore be important for future research to investigate whether age-related differences in trust, including trust in known versus unknown advisors, influence the degree of advice-taking in older adults’ decision making.

### Decision Support Among Older Adults

Older adulthood has been associated with an avoidant decision-making style (Mather, 2006). In comparison to young adults, older adults are more likely to prefer to delegate decision making to others (Finucane et al., 2002), or to completely avoid a decision (Chen et al., 2011). Similarly, Fatima et al. (2020) identified a positive association between age and both dependent and avoidant decision-making styles in a sample of young and middle-aged adults. In the current study, there was no effect of age on advice-taking, suggesting no increased avoidance of decision making with age. The contradictory findings may be attributable to the different methods of assessing decision avoidance. While Fatima et al. assessed how people make “important” decisions using the general decision-making styles self-report questionnaire (Scott & Bruce, 1995), the current study measured behavioral advice-taking in a real but “unimportant” context as a proxy measure of avoidant decision making. However, this explanation seems unlikely given that Delaney et al. (2015) also measured how people make important decisions using the general decision-making styles questionnaire and found that older people were more likely to fit into an independent/self-controlled decision-making style.

The current data, and Delaney et al. (2015), suggest that older adults may not be as avoidant of decision making as previous research has suggested, or that older adults select a decision style depending on the decisional context and available cues (see Mata et al., 2008). Alternatively, it could be argued that older adults were more dependent in the current study by taking as much advice as younger adults despite perceiving the advice to be lower in quality. Taken together, the current data suggest that differing psychological processes may underpin advice-taking across the adult lifespan. The data further suggest that processes influencing perceived advice accuracy (e.g., trust) and decision style should be further investigated to better understand the influence of advice on older adults’ decision making.

### Limitations and Future Directions

The present study did not measure whether people were using emotion regulation during the advice-taking task, but rather their recent use of emotion regulation in everyday life. It can be argued that the methods of emotion regulation that were reported were deliberate strategies, as opposed to more automatic and effortless forms of emotion regulation. The two forms of emotion regulation may be considered as ends on a continuum, where acceptance requires fewer cognitive resources while the change method involves more conscious and explicit attempts to control emotions (Mauss et al., 2007). Furthermore, it has been suggested that as individuals age, deliberative processes decline, and older adults therefore rely more on automatic emotion regulation (Mauss et al., 2007). Further research is needed to understand how deliberate versus automatic emotion regulation processes may contribute to advice-taking in older adulthood. Additionally, older adults in the current study may have been limited by their own memory regarding instances of emotion regulation. Thus, to get an accurate assessment of the interaction between emotion, emotion regulation, and advice-taking in everyday decision making, obtaining real-time data using experience sampling would both relieve memory burden and increase validity.

Given that the perception of advice quality is critical to advice-taking, future research should investigate how age might influence advice-taking from different types of advisors, while manipulating types of emotions experienced, and the types of decision-making scenarios. It should also be noted that because the depressive symptom scores of the present study were clustered around the lower end of the scale, replication of this research with a clinical sample may yield different results. Similarly, given the lack of an association between age and fluid intelligence, the current sample appears to be high functioning and therefore any findings related to age may differ in the general population. Much of the literature on advice-taking, as indexed using the judge–advisor system paradigm, has focused on autonomy-related, information goals, and the desire to improve accuracy (Bailey et al., 2023). Examination of age-related differences in informational versus affiliative goals (Hoppmann & Blanchard-Fields, 2010) is likely to represent an important avenue for future advice-taking research. Finally, as measurements of race/ethnicity/culture, and sociodemographic status were not captured in the current study, we cannot rule out the influence of a sampling bias in the current findings. We recommend that future research examines whether advice-taking differs for older adults from culturally and economically diverse backgrounds.

## Conclusion

Previous research has demonstrated that clinical depression is associated with increased advice-taking (Hofheinz et al., 2017). The current data extend this research by showing that among a nonclinical adult lifespan sample, greater depressive symptoms are associated with increased advice-taking among nonregulators of emotion relative to emotion regulators. These data provide evidence for consistent effects of depressive symptoms on advice-taking across the adult lifespan. A key difference is that older age was associated with perceiving advice to be less accurate. This suggests that, consistent with Bailey et al. (2021), there may be greater vulnerability to taking poor-quality advice in older adulthood.

## Supplementary Material

gbae080_suppl_Supplementary_Material

## Data Availability

This study was preregistered. Data and analytic methods are available at https://osf.io/ymun4/?view_only=502d01b6c15c4389ab548b0038cd87a.
