# Supplementary material for: Cognitive and Emotional Factors Influencing the Incorporation of Advice Into Decision Making Across the Adult Lifespan
Source: J Gerontol B Psychol Sci Soc Sci. 2024 May 13;79(7):gbae080. doi: 10.1093/geronb/gbae080 (PMC11212316; doi:10.1093/geronb/gbae080)
Supplement: gbae080_suppl_Supplementary_Material [file gbae080_suppl_supplementary_material.docx]

***The Journals of Gerontology, Series B: Psychological Sciences and Social Sciences* Supplementary Material: Leon et al. Cognitive and emotional factors influencing the incorporation of advice into decision-making across the adult lifespan.**

Section 1: Additional Methodology Details

Self-Confidence and Perceived Advice Accuracy

Emotions associated with depressive symptoms may also influence advice-taking via self-confidence, which is a metacognitive assessment of the amount and consistency of information in working memory in relation to a judgement (Hütter & Fiedler, 2019). Greater advice-taking has been associated with both lower self-confidence (Hofheinz et al., 2017; Hütter & Fiedler, 2019) and higher self-confidence (Pescetelli et al., 2021). It has been suggested that *low* self-confidence may increase advice-taking in an effort to reduce uncertainty (Duan et al., 2021), while *higher* self-confidence may increase advice-taking due to an attribution error that equates confidence with higher quality advice (Pescetelli et al., 2021). A study involving young adults showed that induced anxiety increased advice-taking, and this was mediated by lower self-confidence (Gino et al., 2012). Our pilot study with a sample independent of the current study (*N* = 132; age range 19-89 years), also showed that lower confidence in a decision was associated with less positive mood (*r* = .25*, p* = .004), but confidence did not influence advice-taking (*r* = -.09, *p* = .307). The associations between emotions associated with depressive symptoms, decision-maker confidence, and advice-taking warrant further investigation.

Participants

Participants were excluded if they reported a neurological condition (such Alzheimer’s disease, stroke, or brain injury, or neuro-atypical disorders).The specific screening questions participants were asked were: (1) “Have you ever been diagnosed with, or experienced a neurological condition (e.g., Alzheimer’s, stroke, brain injury)?”; (2) “Have you ever been diagnosed with, or experienced a mental illness? (e.g., clinical depression, anxiety)”. Participants were then able to describe the condition or illness, and answers were manually screened. There were no exclusions based on reports of clinical depression or anxiety, nor were there any exclusions based on the severity of depressive symptoms. However, we did exclude participants who reported other psychological conditions such as PTSD, bipolar disorder, ADHD, ASD, and personality disorders (n = 10).

Materials and Procedure

*Judge-Advisor Task Practice Phase*

To check for any age group differences in base level knowledge, the practice phase allowed for calculation of opinion difference scores (i.e., initial estimate – actual number of coins in the jar). Following the three practice trials without advice, participants were shown the same images, one at a time, along with their first estimate and advice. After entering a second estimate, participants were presented with the correct number of coins in the jar (range = 71 to 188) to facilitate learning about the task. These images of jars filled with coins differed from those used in the main task.

***Depressive Symptoms***

The depressive subscale of the DASS21 was used. This is a 7-item subscale, as opposed to the full 14-item scale. According to the DASS cut-off scores, Normal = 0-9, Mild = 10-13, Moderate = 14-20, Severe = 21-27, and Extremely severe = 28+. For the DASS21, these scores are halved, and based on these cut-off scores the following participants were classified as severe and extremely severe: Severe: 7 young adults, 4 middle-aged adults, 5 older adults; and extremely severe: 8 young adults, 6 middle-aged adults, 1 older adult. The depressive symptom scores by age group are presented in Supplementary Table 1.

**Supplementary** **Table 1**

*Depressive Symptoms Descriptive Statistics by Age Groups.*

| Age group | M | SD | Min | Max |
| --- | --- | --- | --- | --- |
| Young adults | 6.96 | 6.09 | 0 | 21 |
| Middle-aged adults | 6.13 | 5.31 | 0 | 18 |
| Older adults | 2.97 | 4.12 | 0 | 14 |

***Self-Confidence***

At the end of each judge-advisor round, (round 1 = confidence ratings when no advice had been received (i.e., pre-confidence); round 2 = confidence ratings after receiving advice (i.e., post-confidence)), participants rated how confident they were in their own estimates on a scale from 1 (not at all confident) to 5 (very confident). Participants also rated how accurate they thought the advice was on a scale of 1 (not at all) to 5 (very). For the pre-advice confidence, participants were asked to “rate the following statement on a scale of 1 (not at all) to 5 (very) - I feel confident in my estimates”. For post-advice confidence and perceived accuracy, they were asked to rate the statements “I feel confident in my second estimates”, and "I think that the advice was accurate”. The latter 2 statements were also rated on the same 5-point scale.

**Section 2: Detailed Results and Additional Analyses**

**Results**

Analyses were conducted in *R-Studio* (RStudio Team, 2020) using the rstatix version 0.7.1 (Kassambara, 2021), tidyverse version 1.3.2 (Wickham et al., 2019), Hmisc version 4.7.2 (Harrell, 2022), lme4 version 1.1.3 (Bates et al., 2015), robustlmm version 3.0.4 (Koller, 2016), JWileymisc version 1.3.0 (Wiley, 2022), sjPlot version 2.8.11 (Lüdecke, 2022), nnet version 7.3.19 (Venables & Ripley, 2002), DescTools version 0.99.47 (Signorell et al., 2022), effects version 4.2.2 (Fox & Weisberg, 2019), lmerTest version 3.1.3 (Kuznetsova et al., 2017), and RcmdrMisc (Fox et al., 2023) packages.

**Practice Phase**

A linear model was assessed with age (as a continuous predictor) and practice trial (1, 2, 3) as predictors, and opinion difference as the outcome. The model was significant, *F*(5, 460) = 11.14, *p* < .001, *R^2^* = .11. There was a significant effect of practice trial 3, relative to practice trial 1 (*t* = 2.17, *p* = .030) on opinion difference. This indicates rapid learning among the participants. There was no significant effect of age (*t* = 0.96, *p* = .337), or interactions between age and practice trial 2 relative to age and practice trial 1, or between age and practice trial 3 relative to age and practice trial 1 (*t* = 0.43, *p* = .667, and *t* = 0.18, *p* = .859 respectively).

| Table 2  *Average Opinion Difference by Age Group and Practice Trial*. | | | |
| --- | --- | --- | --- |
| Age group | N | M | SD |
| Young adults | 140 | -41.37 | 80.44 |
| Middle-aged adults | 162 | -30.42 | 61.58 |
| Older adults | 164 | -57.98 | 51.28 |
| Trial 1 | 156 | -70.91 | 74.37 |
| Trial 2 | 155 | -38.32 | 70.41 |
| Trial 3 | 155 | -20.82 | 34.15 |

*Note.* Trial = Practice trial.

***Emotion Regulation Method and Advice-Taking***

To compare change and acceptance methods of regulation as a function of age, we first divided the sample into a young (aged 19-38), middle-aged (aged 40-64), and older (aged 65-88) group. Results of Chi Square tests of independence showed that older adults reported less use of the change method of emotion regulation, compared to the young and middle-aged groups. Middle-aged adults reported less of the acceptance method of emotion regulation, compared to the young and older adults, and young adults reported less use of no emotion regulation method compared to older and middle-aged adults. Inferential statistics are provided in Supplementary Table 6 .

**Emotion Regulation by Depressive Symptoms**

A multinomial logistic regression was performed to examine emotion regulation types (none, accept, change) by the predictor depressive symptoms score. The fit between the intercept only model improved with the addition of the depressive symptoms predictor variable, *X^2^* (2, 3740) = 251.322, Nagelkerke *R^2^* = .141, p < .001. A one-unit increase in depressive symptoms is associated with an increase of reporting the accept type of emotion regulation versus no emotion regulation, by 0.04 (95% CI [ 0.28, 0.34], z = 1.04, *p* = .008). A one-unit increase in depressive symptoms is associated with an increase of reporting the change type of emotion regulation versus no emotion regulation, by 0.16 (95% CI [0.22, 0.27], z = 1.17, *p* < .001).

**Supplementary** **Table 3**

*Age and Depressive Symptom Score Model Comparisons.*

| Sampling Units | | *N* Total Observations = 1698 | | | | | | | |
| --- | --- | --- | --- | --- | --- | --- | --- | --- | --- |
|  |  | *N* Participants = 156 | | | | | | | |
| Random Effects | | Participants = Intercepts | | | | | | | |
|  |  |  | | | | | | | |
| Model specification | Model name | Nested / Simpler model | Fixed Effects added | Model fit | | | | LRT Test against nested | |
|  |  |  |  | AIC | BIC | LL | npar | *df* | *χ*^2^ |
| Predictor main effects | Main effects 1 | - | Age + DSS | 2138.4 | 2165.6 | -1064.2 | 6 |  |  |
| Predictor interaction | Interaction 1 | Main effects 1 | Age × DSS | 2140.4 | 2173.1 | -1064.20 | 6 | 1 | 0.0112 |

*Note*: DSS = Depressive Symptom Score. Age and DSS were grand mean centered. AIC = Akaike’s Information Criteria, BIC = Bayesian Information Criteria, LL = LogLikelihood, LRT = Likelihood Ratio Test, npar = Number of Parameters.

**Supplementary** **Table 4**

*Final Age and Depressive Symptoms Score Model.*

| Fixed Effects | | | | | | | | | |
| --- | --- | --- | --- | --- | --- | --- | --- | --- | --- |
|  | Estimate/Beta | | *SE* | 95% CI | | | *t* | | *p* |
| Intercept | 0.64 | | 0.02 | 0.59, 0.68 | | | 28.68 | | <.001 |
| Age | 0.00 | | 0.00 | -0.00, 0.00 | | | 0.51 | | .612 |
| DSS | 0.01 | | 0.00 | -0.00, 0.01 | | | 1.19 | | .238 |
| Random Effects | | | | | | | | | |
|  | | Variance | | | *SD* | | | ICC | |
| Participant (Intercept) | | 0.06 | | | 0.25 | | | 0.25 | |
| Model Fit | | | | | | | | | |
| R^2^ | | Marginal | | | | Conditional | | | |
|  | | 0.00 | | | | 0.26 | | | |

*Note.* Model equation in R: WOA ~ Age + DSS (1|Participant). DSS = Depressive Symptom Score. Age and DSS were grand-mean centered.

**Supplementary** **Table 5**

*Emotion Regulation and Interactions Model Comparisons.*

| Sampling Units | | *N* Total Observations = 1698 | | | | | | | |  |
| --- | --- | --- | --- | --- | --- | --- | --- | --- | --- | --- |
|  |  | *N* Participants = 156 | | | | | | | |  |
| Random Effects | | Participants = Intercepts | | | | | | | |  |
|  |  | Items = None | | | | | | | |  |
| Model specification | Model name | Nested / Simpler model | Fixed Effects added | Model fit | | | | LRT Test against nested | | |
|  |  |  |  | AIC | BIC | LL | npar | *df* | *χ*^2^ | |
| Predictor main effects | Main effects 1 | - | Age + DSS + ER | 2139.6 | 2172.2 | -1063.8 | 6 |  |  | |
| 2-way interaction | Interaction 1 | Main effects 1 | DSS + (Age × ER) | 2141.4 | 2179.5 | -1063.7 | 7 | 1 | 0.1737 | |
| 2-way interaction | Interaction 2 | Main effects 1 | Age + (DSS × ER) | 2131.1 | 2169.1 | -1058.5 | 7 | 1 | **10.521**** | |
| 2-way interaction | Interaction 3 | Main effects 1 | ER + (Age × DSS) | 2141.6 | 2179.7 | -1063.8 | 7 | 1 | 0.9718 | |
| 3-way interaction | Interaction 4 | Interaction 2 | Age × DSS × ER | 2135.5 | 2189.9 | -1057.8 | 10 | 3 | 0.6661 | |

*Note*: DSS = Depressive Symptom Score. ER = Emotion Regulator (baseline is ‘No’). Age and DSS were grand mean centered. AIC = Akaike’s Information Criteria, BIC = Bayesian Information Criteria, LL = LogLikelihood, LRT = Likelihood Ratio Test, npar = Number of Parameters.

**Supplementary** **Table 6**

*Contingency table of frequencies between each age group and emotion regulation method.*

| Age group | Change | Acceptance | None |
| --- | --- | --- | --- |
| Young | 53% | 28% | 19% |
| Middle | 39% | 22% | 39% |
| Older | 33% | 29% | 38% |

**Supplementary** **Table 7**

*Intercorrelations between age, depressive symptoms, effort exerted to change emotions, pre- and post-advice confidence, perceived advice accuracy, fluid IQ, and average weight of advice among participants who reported the change method (n =* 64*).*

| Variable | **1** | **2** | **3** | **4** | **5** | **6** | **7** | **8** |
| --- | --- | --- | --- | --- | --- | --- | --- | --- |
| 1. Age | - |  |  |  |  |  |  |  |
| 2. DSS | -0.14 | - |  |  |  |  |  |  |
| 3. Change effort | -0.19 | 0.38* | - |  |  |  |  |  |
| 4. Pre-confidence | -0.12 | 0.09 | 0.00 | - |  |  |  |  |
| 5. Post-confidence | -0.08 | 0.08 | -0.01 | 0.63 | - |  |  |  |
| 6. PAA | -0.38* | 0.22 | 0.22 | 0.33* | 0.46** | - |  |  |
| 7. Fluid IQ | -0.07 | -0.28 | -0.22 | -0.06 | 0.02 | -0.15 | - |  |
| 8. WOA | 0.12 | -0.15 | 0.06 | -0.01 | -0.02 | 0.25 | 0.00 | - |

*Note*: DSS = Depressive symptom score; Change effort = Effort exerted to change emotions on a scale from 1 (no effort at all) to 10 (the most effort); PAA = Perceived advice accuracy; WOA = average WOA; * indicates *p* < .05. ** indicates *p* < .01.

**Supplementary** **Table 8**

*Intercorrelations between age, depressive symptoms, emotion regulation effort for acceptance, pre- and post-advice confidence, perceived advice accuracy, fluid IQ, and average weight of advice among participants who reported the accept method (n =* 41*).*

| Variable | **1** | **2** | **3** | **4** | **5** | **6** | **7** | **8** |
| --- | --- | --- | --- | --- | --- | --- | --- | --- |
| 1. Age | - |  |  |  |  |  |  |  |
| 2. DSS | -0.47* | - |  |  |  |  |  |  |
| 3. Accept effort | -0.18 | 0.34 | - |  |  |  |  |  |
| 4. Pre-confidence | -0.06 | -0.17 | 0.13 | - |  |  |  |  |
| 5. Post-confidence | -0.23 | 0.14 | 0.20 | 0.65 | - |  |  |  |
| 6. PAA | -0.24 | 0.32 | 0.12 | 0.41 | 0.55** | - |  |  |
| 7. Fluid IQ | 0.14 | -0.15 | -0.16 | -0.29 | -0.22 | -0.23 | - |  |
| 8. WOA | -0.12 | -0.11 | -0.03 | -0.31 | -0.15 | 0.06 | 0.08 | - |

*Note*: DSS = Depressive symptom score; Accept effort = Effort exerted to accept emotions on a scale from 1 (no effort at all) to 10 (the most effort); PAA = Perceived advice accuracy; WOA = average WOA; * indicates *p* < .05. ** indicates *p* < .01.

**Supplementary** **Table 9**

*Intercorrelations between age, depressive symptoms, pre- and post-advice confidence, perceived advice accuracy, fluid IQ, and average weight of advice among participants who reported no emotion regulation (n* = 51*).*

| Variable | **1** | **2** | **3** | **4** | **5** | **6** | **7** |
| --- | --- | --- | --- | --- | --- | --- | --- |
| 1. Age | - |  |  |  |  |  |  |
| 2. DSS | -0.27 | - |  |  |  |  |  |
| 3. Pre-confidence | -0.17 | -0.05 | - |  |  |  |  |
| 4. Post-confidence | -0.24 | -0.15 | 0.82 | - |  |  |  |
| 5. PAA | -0.07 | 0.14 | 0.35 | 0.43* | - |  |  |
| 6. Fluid IQ | 0.04 | -0.21 | -0.17 | -0.07 | -0.12 | - |  |
| 7. WOA | -0.02 | 0.44* | -0.20 | -0.18 | 0.32 | -0.14 | - |

*Note*: DSS = Depressive symptom score; PAA = Perceived advice accuracy; WOA = average WOA; * indicates *p* < .05.

**Supplementary** **Table 10**

*Exploratory Models.*

| Sampling Units | | *N* Total Observations = 1698 | | | | | | | |
| --- | --- | --- | --- | --- | --- | --- | --- | --- | --- |
|  |  | *N* Participants = 156 | | | | | | | |
| Random Effects | | Participants = Intercepts | | | | | | | |
|  |  |  | | | | | | | |
| Model specification | Model name | Nested / Simpler model | Fixed Effects added | Model fit | | | | LRT Test against nested | |
|  |  |  |  | AIC | BIC | LL | npar | *df* | *χ*^2^ |
| Predictor main effects | Baseline | - | ERM + Age + Fluid IQ + DSS + PC + PAA | 2128.5 | 2182.9 | -1054.2 | 10 |  |  |
| Age × DSS interaction | Interaction 1 | Baseline | (Age × DSS) + ERM + Fluid IQ + PC + PAA | 2130.5 | 2190.3 | -1054.2 | 11 | 1 | 0.0185 |
| ERM × DSS interaction | Interaction 2 | Baseline | (ERM × DSS) + Age + Fluid IQ + PC + PAA | 2120.0 | 2185.3 | -1048.0 | 12 | 2 | **12.459**** |
| ERM × DSS × Fluid IQ interaction | Interaction 3 | Interaction 2 | (ERM × DSS × Fluid IQ) + Age + PC + PAA | 2123.4 | 2215.8 | -1044.7 | 17 | 5 | 6.6238 |
| PC × DSS interaction | Interaction 4 | Interaction 2 | (ERM × DSS) + (DSS × PC) + Age + Fluid IQ + PAA | 2121.9 | 2192.6 | -1047.9 | 13 | 1 | 0.1549 |
| ERM × DSS × Age interaction | Interaction 5 | Interaction 2 | (ERM × DSS × Age) + Fluid IQ + PC + PAA | 2126.0 | 2218.4 | -1046.0 | 17 | 5 | 4.0610 |
| ERM × DSS × PAA interaction | Interaction 6 | Interaction 2 | (ERM × DSS × PAA) Age + Fluid IQ + PC | 2127.4 | 2219.8 | -1046.7 | 17 | 5 | 2.6464 |
| PC × PAA interaction | Interaction 7 | Interaction 2 | (ERM × DSS) + Age + Fluid IQ + (PC × PAA) | 2121.2 | 2191.9 | -1047.6 | 13 | 1 | 0.8316 |
| PC × Age interaction | Interaction 8 | Interaction 2 | (ERM × DSS) + Fluid IQ + (PC × Age) + PAA | 2121.8 | 2192.5 | -1047.9 | 13 | 1 | 0.2123 |
| Age × Fluid IQ interaction | Interaction 9 | Interaction 2 | (ERM × DSS) + (Age × Fluid IQ) + PC + PAA | 2119.5 | 2190.2 | -1046.8 | 13 | 1 | 2.5454 |

*Note*: DSS = Depressive Symptom Score, ERM = Emotion Regulation Method (Change or Accept, compared to None), PC = Pre-advice Confidence, PAA = Perceived Advice Accuracy. Continuous variables were grand mean centered. AIC = Akaike’s Information Criteria, BIC = Bayesian Information Criteria, LL = LogLikelihood, LRT = Likelihood Ratio Test, npar = Number of Parameters.

**Supplementary** **Table 11**

*Final Exploratory Model.*

| Fixed Effects | | | | | | | | | |
| --- | --- | --- | --- | --- | --- | --- | --- | --- | --- |
|  | Estimate/Beta | | *SE* | 95% CI | | | *t* | | *p* |
| Intercept | 0.75 | | 0.04 | 0.67, 0.84 | | | 17.08 | | **<.001** |
| Age | 0.00 | | 0.00 | -0.00, 0.00 | | | 1.33 | | .185 |
| DSS | 0.03 | | 0.01 | 0.01, 0.04 | | | 2.96 | | .**003** |
| ERM (Accept) | -0.01 | | 0.06 | -0.13, 0.12 | | | 0.04 | | .965 |
| ERM (Change) | -0.01 | | 0.06 | -0.13, 0.10 | | | 0.21 | | .838 |
| Fluid IQ | -0.00 | | 0.01 | -0.03, 0.03 | | | 0.09 | | .926 |
| PC | -0.08 | | 0.02 | -0.13, -0.03 | | | 3.24 | | **.001** |
| PAA | 0.11 | | 0.03 | 0.06, 0.17 | | | 4.30 | | **<.001** |
| DSS× ERM (Accept) | -0.03 | | 0.01 | -0.05, -0.01 | | | 2.47 | | **.014** |
| DSS × ERM (Change) | -0.04 | | 0.01 | -0.06, -0.01 | | | 3.19 | | **.001** |
| Random Effects | | | | | | | | | |
|  | | Variance | | | *SD* | | | ICC | |
| Participant (Intercept) | | 0.07 | | | 0.27 | | | 0.57 | |
| Model Fit | | | | | | | | | |
| R^2^ | | Marginal | | | | Conditional | | | |
|  | | 0.13 | | | | 0.63 | | | |

*Note.* Model equation in R: WOA ~ Age + DSS*ERM + Fluid IQ + PC + PAA + (1|Participant). DSS = Depressive Symptom Score, ERM = Emotion Regulation Method (Change or Accept, compared to None), PC = Pre-advice Confidence, PAA = Perceived Advice Accuracy. Continuous variables were grand mean centered.
